# Why Animal‐Source Foods Are not Consumed by Women and Children in the Amhara Region: A Qualitative Study

**DOI:** 10.1111/mcn.70006

**Published:** 2025-02-26

**Authors:** Netsanet Fentahun, Valerie Flax, Yeshalem Mulugeta

**Affiliations:** ^1^ Department of Nutrition and Dietetics, School of Public Health, College of Medicine and Health Sciences Bahir Dar University Bahir Dar Ethiopia; ^2^ RTI International Research Triangle Park North Carolina USA

**Keywords:** animal source foods, children, Ethiopia, lactating women, pregnant women

## Abstract

This study aimed to understand the barriers and facilitators of animal source food (ASF) consumption among women and children in Wag Himra, South Gondar, and Central Gondar zones of Amhara Region, Ethiopia. Women and men with children under 2 years, grandmothers, community and religious leaders, and midwives and health workers were included. Thirty separate focus group discussions and 35 key informant interviews were conducted from July to August 2022. To ensure the quality of qualitative research findings, the criteria of trustworthiness were maintained by the following: credibility, transferability, dependability, and conformability. Qualitative thematic analysis was carried out using Qualitative Data Analysis Miner. Barriers to children's ASF consumption were poverty, age of the child, illness, high cost of ASFs, loss of parents, divorcee, and a lack of livestock. For pregnant and lactating women, the main barriers to ASF consumption were lack of animals in the household, financial constraints, illness, fear of having a big baby, religious fasting, and morning sickness. Family affluence, the availability of ASFs in the household, liking the taste of ASFs, and receiving nutrition education on ASFs were facilitators for children and pregnant and lactating women to consume ASFs. Barriers to ASF consumption in Amhara Region include factors related to livelihoods and social norms or beliefs. Programmes could offer a combination of livelihood supports and social and behaviour change communication to increase ASF consumption by women and children.

## Summary

- The pregnant and lactating women considered animal‐source foods as food for holidays and societal events.
- The high cost of animal‐source foods and financial constraints within households discouraged pregnant and lactating women and children from consuming animal‐source foods.
- Family affluence, the availability of ASFs in the household, liking the taste of animal‐source foods, and receiving nutrition education on ASFs were factors that facilitated the consumption of ASFs by children and pregnant and lactating women.

## Graphical abstract

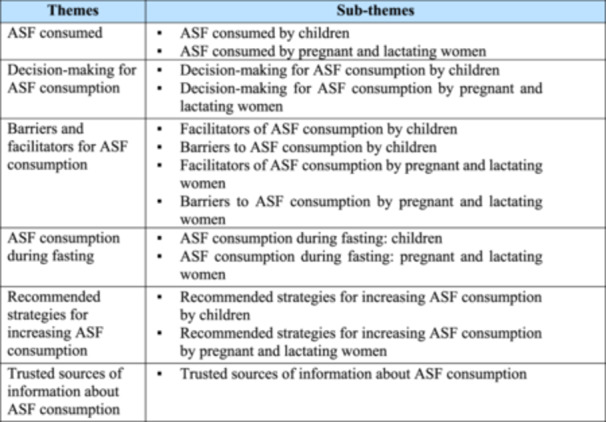

## 1 Introduction

Maternal, infant, and young child nutrition in the first 1000 days of life plays a significant role in optimal child growth, brain development, health, and survival (World Health Organization 2014). Frequency and diversity of meals consumed by women during pregnancy and by young children are fundamental for child growth and development. Animal source foods (ASFs) include milk, yogurt, and cheese (dairy products); meat, fish, poultry, and organ meats (flesh foods), and eggs. ASFs account for three out of eight food groups in children's dietary diversity recommended by the World Health Organization (WHO 2023) and three out of ten food groups in women's dietary diversity (Hanley‐Cook et al. 2020). ASFs are an important source of protein and bioavailable micronutrients, including zinc, iron, calcium, vitamin A, folic acid, vitamin B12, and riboflavin (Schmid and Walther 2013; Melse‐Boonstra 2020; Miller et al. 2022). Additionally, ASFs contain lower levels of anti‐nutritional factors, such as phytate, which inhibit iron and zinc absorption, compared to plant source foods (Grace et al. 2018).

Adequate consumption of ASFs is associated with improved growth, development, levels of physical activity, pregnancy outcomes, and health (Neumann et al. 2007; Haileselassie et al. 2020). ASFs can improve children's nutritional status, (Jin and Iannotti 2014) because they provide essential macro and micronutrients (Dror and Allen 2011). Children who consume ASFs have a lower risk of being stunted and wasted compared to their counterparts who do not consume ASFs (Headey 2018; Eaton et al. 2019).

Despite the importance of ASFs in averting malnutrition, consumption of ASFs is inadequate in Ethiopia. Although Ethiopia is among the countries with the largest livestock population globally, animal products are primarily used as sources of cash for households rather than including them in daily meals (Tafere and Hassen 2012; Gernand et al. 2016; Zerfu et al. 2016). Consequently, Ethiopians eat cereal‐based monotonous diets with infrequent consumption of ASFs, fruits, and vegetables (Abegaz et al. 2018; Nana and Zema 2018). Furthermore, although livestock possession is most common in rural areas, the per capita consumption of ASFs is much higher in urban areas compared with rural settings (Abegaz et al. 2018). In rural areas, ASFs are normally consumed during holidays and social events because ASFs are considered a food for special occasions and are not eaten regularly (Tafere and Hassen 2012). Despite its significant role in preventing macro and micronutrient deficiencies, ASF consumption is still a major challenge in Ethiopia. The barriers that hinder and factors that enable ASF consumption are context‐specific and socio‐culturally sensitive and understanding context‐specific factors is very important to improve ASF consumption (Fite et al. 2022). However, there is limited evidence on these factors in the Amhara Region, Ethiopia. To address this gap, this study aimed to understand the barriers that hinder and factors that enable ASF consumption among women and children in the Amhara Region as part of the formative research for a United States Agency for International Development (USAID)‐funded programme.

## 2 Methods

### 2.1 Study Setting

This study was conducted during the first year of the USAID Bureau for Humanitarian Affairs Poverty Reduced Sustainably in an Environment of Resilient and Vibrant Economy (PReSERVE) project. PReSERVE is implemented by Food for the Hungry and partners in the Amhara Region in 11 of the 70 woredas (districts) targeted by the Ethiopian government's Productive Safety Net Programme. PReSERVE aims to improve food security by supporting production of ASFs and other nutrient‐rich foods, increasing access to savings and credit, providing access to clean water and sanitation, and improving skills and employment opportunities for youth.

This study was conducted in Sahila in Wag Himra zone; Simada, Tach Gayint, and Lay Gayint in South Gondar zone; and East Belessa in Central Gondar zone. These woredas are in three zones: Sahila in the lowland; Simada, Tach Gayint, and Lay Gayint in the predominantly highland; and East Belessa in the largely lowland Central Gondar zone. Cereal crops are grown and livestock, including cattle, sheep, and goats, are raised, but the mix of crops and livestock varies by zone and woreda. These woredas are characterised by high levels of malnutrition, cyclical drought, erratic rainfall and flooding, and other drivers of vulnerability associated with climate change.

### 2.2 Study Design and Period

This formative qualitative study was conducted to explore barriers that hinder and factors that enable ASF consumption among women and children in Amhara Region, Ethiopia from July to August 2022.

### 2.3 Study Population

Women with children under 2 years and men as husbands/partners or fathers were invited to participate in focus group discussions (FGDs). The FGDs were held separately for women and men. Grandmothers, community and religious leaders, midwives and health workers were included in key informant interviews (KIIs).

### 2.4 Sample Size Determination and Sampling Technique

Sample sizes for qualitative research can vary, but researchers consider the scope of the study, the nature of the topic (i.e., complexity, accessibility), the quality of the data, the study design, and the information saturation parameter while determining the sample. A total of 30 FGDs (6 per woreda) and 35 KIIs (7 per woreda) were conducted. From the selected sample woredas, 20% of kebeles (wards) were included in this study (3 kebeles for each woreda). FGD participants were selected using a homogeneous sampling technique (Jager et al. 2017). Homogenous sampling technique involves selection of homogenous or closely related units or participants (e.g., women with a child under 2 years) to give a detailed picture of a particular phenomenon among people with a common identity and to discuss their shared experiences (Jager et al. 2017). Key informants were selected purposively based on their experiences, involvement in health care provision, position/status, and working area (Ahmed 2024). FGD and KII participants were accessed through kebele officials and health extension workers.

### 2.5 Data Collection Methods

FGDs and KIIs were conducted using semi‐structured FGD and interview guides. The semi‐structured questions used for the focus groups and interviews focused on the types of ASFs given to children under 24 months and eaten by pregnant/lactating women, household decision‐making related to ASF consumption, factors that limit the consumption of ASFs by children and pregnant/lactating women, factors that facilitate the consumption of ASFs by children and pregnant/lactating women, strategies for the could increase ASF consumption by children and pregnant/lactating women, and trusted sources of information or advice regarding decisions about ASF consumption.

Six professionals (two in each session) facilitated FGDs, and three interviewers conducted the KII. The FGDs facilitators and KII interviewers were experts with master's degrees in public health nutrition or public health who are fluent in the local language and have previous experience in collecting qualitative data. On average, the FGDs and interviews took 1 h. The FGDs and KIIs were digitally recorded, and field notes were taken. The FGDs were held separately for women and men. Each FGD had 10 participants.

### 2.6 Data Quality Assurance

The trustworthiness of the findings were ensured by following the criteria of credibility, transferability, dependability, and conformability (Connelly 2016). Credibility: Before data collection, the research team and experts evaluated the content of both FGD and KII guides. Professionals who had qualitative data collection experience and proficiency in local language collected the qualitative data. The research technical team supervised the overall data collection. Transferability: The research team used probes to get more details, when needed, and summarised the emerging issues in one section before starting another section of the guide. Participants were given a chance to comment on the summary if they felt it did not sufficiently represent what they said. Dependability: The research team reviewed the preliminary findings to correct errors and interpretations that did not align with the data. Conformability: The research team observed and documented participants' body language, expressions, and tone of voice during the FGDs and KIIs.

### 2.7 Data Processing and Analysis

The research team reviewed the field notes and listened to the audio recordings repeatedly to understand the participants' responses. The recordings were transcribed verbatim in the Amharic language and then translated from Amharic to English. Transcripts were entered into the QDA MINER LITE version 3.1 software for qualitative content analysis (Hsieh and Shannon 2005). Codes were applied to strings of text representing similar ideas using the software and cross‐checked between researchers. Codes were then organised into themes. Finally, the results were presented narratively with illustrative quotations.

### 2.8 Ethical Statement

This study was conducted according to the guidelines laid down in the Declaration of Helsinki and all procedures involving research study participants were approved by The Institutional Review Board of Bahir Dar University. Written informed consent was obtained from all subjects. The objective of the project was disclosed to the respondents. The data were collected anonymously to maintain confidentiality. The respondents' autonomy was ensured by telling them that they could stop participating at any time.

## 3 Results

The average age of the FGD participants was 26 years and ranged from 18 to 58 years. The average age of participants in the key informant interviews was 33 years and ranged from 22 to 65 years. The majority of the respondents were not able to read and write. A total of 35 KIIs (17 men and 18 women) and 30 FGDs with 300 participants (150 women and 150 men) were conducted. Table 1 shows the summary of the themes and domains related to ASF consumption among women and children in Amhara Region. A total of 6 themes and 13 sub‐themes were identified.

**Table 1:** Summary of themes and codes related to ASF consumption among women and children in Amhara Region 2022.

Themes	Sub‐themes
ASF consumed	ASF consumed by children ASF consumed by pregnant and lactating women
Decision‐making for ASF consumption	Decision‐making for ASF consumption by children Decision‐making for ASF consumption by pregnant and lactating women
Barriers and facilitators for ASF consumption	Facilitators of ASF consumption by children Barriers to ASF consumption by children Facilitators of ASF consumption by pregnant and lactating women Barriers to ASF consumption by pregnant and lactating women
ASF consumption during fasting	ASF consumption during fasting: children ASF consumption during fasting: pregnant and lactating women
Recommended strategies for increasing ASF consumption	Recommended strategies for increasing ASF consumption by children Recommended strategies for increasing ASF consumption by pregnant and lactating women
Trusted sources of information about ASF consumption	Trusted sources of information about ASF consumption

### 3.1 ASF Consumption

This theme included ASFs consumed by children and pregnant and lactating women.

#### 3.1.1 ASF Consumption by Children

Participants stated that the children did not consume ASFs. They only consumed ASFs during the holidays, such as Easter, New Year, and Christmas. Additionally, the majority of participants did not prioritise children when serving ASFs to family members.

We do not give ASFs regularly and with priority to children. We feed ASFs to children during holidays; otherwise, we give cereals with legumes. We give priority in order of age: older first and then the younger children.

FGD, male, Tach Gayint

The local culture gives priority to men over women and older children over younger children when serving ASFs.

Currently, there is no ASF, but when it is available, we give priority to men and older children. We give a large amount to them.

FGD, male, East Belesa

#### 3.1.2 ASF Consumption by Pregnant and Lactating Women

Participants stated that pregnant and lactating women consumed foods that are available at home. Like other family members, pregnant and lactating women consumed ASFs during the holidays. Priority was not given to pregnant and lactating women when serving ASFs to family members.

Currently, there is a shortage of ASFs. All family members, including pregnant and lactating women, eat ASFs only during the holidays.

FGD, female, Sahila

If ASFs are available, pregnant women eat milk, butter, meat, and eggs, but currently we eat what we have at home, ‘Bet Yaferawun in Amharic.’ We are at the end of our scarce food resources (spoken with sorrow). Previously, when we gave birth, we ate eggs and meat from sheep, goats, and hens, but now we struggle to eat even Injera [fermented bread made from teff or other grains] with Shiro [stew made from legume flour, oil, onions pureed, water, berbere spice and salt].

FGD, female, Tach Gayint

### 3.2 ASF Consumption Decision‐Making

This category included decision‐making for ASF consumption by children and pregnant and lactating women.

#### 3.2.1 Decision‐Making About Children's Consumption of ASFs

The study participants reported that both the mother and father jointly decided about ASF consumption for their children.

There is no difference between mother and father on the decision for our household issues. We decide as a family to feed our child ASFs.

FGD, female, Sahila

The older generation, including me, loves our mothers because mothers care so much. Now, the current generations love their fathers because fathers started to care for their children. So, fathers and mothers decide which ASF is given to their children.

KII, grandmother, Lay Gayint

#### 3.2.2 Decision‐Making About Pregnant and Lactating Women's Consumption of ASFs

The study participants raised different points regarding decision‐making about pregnant and lactating women's consumption of ASFs. More than half of the study participants explained that husbands decided about ASF consumption for pregnant and lactating women. Sometimes, both women and their husbands were responsible for making decisions regarding women's ASF consumption.

Men decide what their wives eat or not because men make decisions about how money is spent in the home. Males purchase ASFs for household consumption, and I prepare them for consumption.

FGD, female, East Belesa

The men and women together decide whether it is better or not to give some ASFs to pregnant and lactating women, or which task must be performed first, and which task should be performed last, starting from market up to farmland decisions.

KII, community leader, Lay Gayint

### 3.3 Barriers and Facilitators for ASF Consumption

This theme included barriers to ASF consumption by children and pregnant and lactating women and facilitators of ASF consumption by children and pregnant and lactating women.

#### 3.3.1 Barriers to ASF Consumption by Children

Poverty, age of the child, illness, high cost of ASFs, loss of parents, divorcee, and lack of livestock were the most common barriers to ASF consumption by children. Lack of animals in the household was described by participants as the primary determinant of ASF consumption among children under 2 years. Participants explained that the majority of community members do not possess animals like oxen, milk cows, and sheep due to a shortage of animal feed and water, which inhibits consumption of ASFs.

God does not allow us to eat ASFs. We live in desert areas that have no grass or water for animals. As a result, we do not have animals, which in turn inhibits us from giving ASFs to children. So ASF consumption is not God's will (‘Egziabher yalfeterewun keyet enamta’ in Amharic) here.

FGD, female, Sahila

Poverty was another barrier that limited ASF consumption by children. All participants mentioned affordability and scarcity as barriers that limit ASF consumption by children.

We do not give ASFs to children due to a lack of money to buy ASFs.

FGD, female, Simada

We are so poor. We live in a drought area. We know about a balanced diet. We learn in theory, but there is no practice because of poverty.

KII, Women's Development Army, Sahila

Age was one of the barriers that limit the ASF consumption of children. Study participants stated that meat was not given to children under 2 years of age.

The age of the child determines the consumption of ASF. We do not give meat to under 2‐year‐old children.

FGD, female, Sahila

#### 3.3.2 Facilitators of ASF Consumption by Children

Possession of livestock at the home, household wealth, and receiving nutrition education on ASFs through the health system and through nongovernmental organisations were facilitators to feeding ASFs to children. The wealthiest families and households that possessed livestock gave ASFs to children.

The presence of animals at home encourages us to feed ASFs to our children. If we get yogurt and meat, we feed our children with them.

FGD, female, Simada

The presence of health education given by health extension workers, health developmental army, and other health care workers facilitates the consumption of ASFs.

KII, grandmother, Lay Gayint

#### 3.3.3 Barriers to ASF Consumption by Pregnant and Lactating Women

Study participants mentioned environmental conditions (such as drought), lack of animals in the household, financial constraints, illness, fear of having a big baby, fasting, and morning sickness as barriers that limited ASF consumption by pregnant and lactating women. The community recommended avoiding ASF consumption during pregnancy due to the perception that ASFs increase intrauterine growth leading to a big baby, which could result in obstructed labour. However, the community recommended ASF consumption for lactating women to replace their damaged tissue.

ASFs are not allowed during pregnancy because they can increase weight and make delivery difficult. But after delivery it can be given, and during childbed period, women can consume milk and meat.

FGD, male, East Belesa

The following barriers hinder us from consuming ASFs. Those barriers are lack of resources, illness, environmental conditions, workload, and lack of access to ASFs at home. We are even forced to sell the animal products that we have to exchange for other foodstuffs that are vital for household consumption, such as legumes and cereals.

FGD, female, Sahila

The major reason is drought. There is no food for the animals, especially this year. No animals were available this year. They died because of drought.

KII, Women's Development Army, Sahila

#### 3.3.4 Facilitators of ASF Consumption by Pregnant and Lactating Women

There are different facilitators that encourage ASF consumption among pregnant and lactating women. Those facilitators are perceptions related to health, household wealth, availability of ASFs in the household, and preference for ASFs. Moreover, health care workers and agricultural workers encourage pregnant and lactating women to consume ASFs.

Availability of animals like milking cows, sheep, and chicken in our family enables pregnant and lactating women to consume whatever we need based on the education that we get from health extension workers. Resource availability was another critical factor. If we had resources, we could feed ASFs to children and pregnant and lactating women.

FGD, male, Lay Gayint

The availability of health education, the presence of support from agriculture officers and health care workers, and the tradition of helping one another, particularly visiting mothers in the postpartum period, facilitate consumption of ASFs.

KII, community leader, Tach Gayint

Factors that facilitate consumption of ASFs by pregnant or lactating women are the presence of support from the government and improved gender equality.

KII, Women's Development Army, Tach Gayint

### 3.4 ASF Consumption During Fasting

This theme is related to ASF consumption during the fasting season (period when ASF consumption is restricted among Orthodox Christians) by the community, children, and pregnant and lactating women.

#### 3.4.1 Children's ASF Consumption During Fasting

The majority of the study participants claimed that fasting had no effect on the ASF consumption of young children. Children are obliged to start fasting at the age of seven. Children less than 7 years old are allowed to consume milk and eggs if available in the household during fasting periods, but meat is not available. The community restricts the provision of ASFs to children during the fasting season due to fear of contamination of cooking utensils used to prepare ASFs.

Cooking ASFs contaminates food dishes and the smell of ASFs leads to breaking religious rules. Due to these beliefs, children are not allowed to eat ASFs during fasting. Even though some community members are willing to give ASFs to their children, ASFs are not available during the fasting period.

FGD, male, East Belesa

Children whose age is greater than 7 years and pregnant and lactating women are fasting. They do not eat both eggs and meat. But they eat foods other than ASFs.

KII, health extension worker, East Belesa

#### 3.4.2 Pregnant and Lactating Women's ASF Consumption During Fasting

Study participants claimed that pregnant women did not consume ASFs during the fasting period. They are obliged to abstain from taking ASFs during fasting. After delivery, the religion/culture allows women to consume ASFs until the infant's baptism. Currently, the Ethiopian Orthodox Christian Church is teaching pregnant and lactating women to eat ASFs, but they still do not consume them.

During pregnancy we don't eat ASFs at the time of the fasting period, but the religion allows us to eat ASFs until 2 months after delivery to repair our body. When we give birth during a fasting period, we (‘Emechatochu’ in Amharic) eat eggs, meat, and milk.

FGD, female, Tach Gayint

Pregnant mothers fast the entire pregnancy period, but lactating women eat ASFs until the baptism day (40 days for male and 80 days for female babies) of their postnatal period if it is a fasting period. We do order no fasting until 6 months, when the priest may order fasting to compensate for the breaking of the fasting law during the postnatal period.

KII, religious leader, Lay Gayint

### 3.5 Recommended Strategies for Increasing ASF Consumption

This theme described the recommended strategies to improve the ASF consumption of children and pregnant and lactating women.

#### 3.5.1 Recommended Strategies for Increasing ASF Consumption of Children

Financial assistance, the provision of animals, such as chickens, goats, lambs, and cows, and nutrition instruction were frequently mentioned as effective ways to increase children's consumption of ASF.

Distributing the best animal breeds to the community, increasing their quality, and giving health education would be effective strategies for increasing ASF consumption.

KII, community leader, Sahila

Savings/loan, community cooperation, increasing number and quality of livestock will be effective for increasing ASF consumption.

KII, grandmother, Lay Gayint

Improving health promotion programmes, increasing partner support, creating job opportunities, and improving cow milk production will be other effective strategies for increasing ASF consumption by women and children.

KII, midwife, Simada

#### 3.5.2 Recommended Strategies for Increasing ASF Consumption of Pregnant and Lactating Women

Domestic animals, animal feed, and the provision of starting cash were commonly mentioned as suggested measures to increase ASF consumption by pregnant and lactating women.

Increasing better genetic cattle production and uninterrupted governmental support will be effective strategies to increase ASFs for pregnant or lactating women's consumption.

KII, Women's Development Army, Tach Gayint

Increase awareness towards the eating habits of ASF rather than selling to market, increase the working habits of the community, expand health education, advocate good governance, create job opportunities for youths, and sustainable organisational support will improve ASFs for pregnant or lactating women's consumption.

KII, community leader, Simada

### 3.6 Trusted Sources of Information About ASF Consumption

This category described the trusted sources of information about ASF consumption by the community, children, and pregnant and lactating women.

#### 3.6.1 Trusted Sources of Information About ASF Consumption

The community receives information about ASF consumption from religious leaders, health development army, neighbours, health professionals, radio, and grandparents. Information from professionals and religious leaders is considered reliable and trusted, but the community distrusts nutrition information given by the health development army. Because the majority of the health development army is not educated, the community mistrusts them.

We get reliable information from health professionals and religious leaders. Then we decide based on the information and advice we get from them. They are the most trusted source of information in our community. We prefer health professionals’ and religious leaders’ advice. We get information from religious leaders, which is important for decision‐making regarding ASF consumption. We also received information from the health development army, but we don't mind them.

FGD, female, Lay Gayint

First they trust religious leaders, second they trust community leaders, and third they trust health professionals.

KII, nutrition focal person, East Belesa

## 4 Discussion

In the study area, the community considered ASFs as a food for holidays and societal events. Because of this, ASFs were not included in the diet of most households out of the holidays. As a result, children and pregnant and lactating women rarely consumed ASFs, like other household members. The finding that ASFs were mainly consumed during holidays diverged from a previous study in Ethiopia, in which rural households in Ethiopia did not consume ASF on regular basis (Daba et al. 2021). Limited availability, high price of ASFs, and requirements of fasting periods are the likely reasons for not consuming ASFs.

Regarding the intra‐household allocation of ASFs, men were served first followed by older children. Younger children and pregnant and lactating women were served last and given small portions. Studies in Kenya and Timor‐Leste also showed that men are served first and given large portions of ASFs as compared with other family members (Bonis‐Profumo et al. 2022; Bukachi et al. 2022). This gender bias in ASF allocation, especially for pregnant and lactating women and children, should inform the design of programmes designed to tackle malnutrition in the Amhara Region.

There is evidence that women's participation in household decision‐making, including food purchases, is correlated with household consumption of a wider variety of foods, including ASFs (Amugsi et al. 2016; Wong et al. 2018). However, in this study, husbands tended to make decisions about ASF consumption by pregnant and lactating women. Although mothers and fathers jointly decided about ASF consumption for their children, fathers control household finances related to more expensive food purchases, which limits ASF provision to children in the study area. This male dominance in deciding about ASF utilisation of pregnant and lactating women and children might have negative impacts on nutrition outcomes of women and children.

Research from Ethiopia and other low‐ and middle‐income countries align with many of our findings related to facilitators and barriers to ASF consumption. The presence of the health extension programme facilitated the consumption of ASFs by the community. Similar to this finding, a previous study in Ethiopia showed health extension workers' positive role in encouraging consumption of ASFs to children (Ayana et al. 2017; Haileselassie et al. 2020). This may be related to the health extension workers' provision of health and nutrition services, such as training and counselling, to the community.

Alive & Thrive is an example of a successful programme in Amhara that offered thorough behaviour change communication, including interpersonal communication, community mobilisation, and mass media, together with nutrition‐sensitive agricultural activities to improve complementary feeding practices and reduce child stunting (Kim et al. 2016; Kim et al. 2019). However, there is still much work to be done to alleviate household food insecurity and other challenges, which will make it possible for households in Amhara to carry out recommended practices supported by behaviour change communication activities.

The high cost of ASFs and financial constraints within households discouraged the community and children from consuming ASFs. Most of the households could not afford ASFs in the local market, such as eggs, chicken, butter, and meat, due to their high price and low level of household income. This result was consistent with previous studies that showed low ASF consumption was due to high cost of ASFs and financial constraints (Haileselassie et al. 2020; Daba et al. 2021).

Livestock ownership was a facilitator for ASF consumption by children and pregnant and lactating women because it improves availability of ASFs at the household level (Azzarri et al. 2015). Lack of livestock ownership limited ASF consumption by children, pregnant and lactating women, and the community. This finding aligns with a study in Ethiopia that reported lack of livestock ownership as a barrier that hinders consumption of ASFs (Daba et al. 2021). Age of the child was another barrier for ASF consumption by children. In the study area, meat was not given to small children. Previous studies in Ethiopia showed higher odds of ASF consumption among older children compared with younger children and infants (Headey et al. 2018; Potts et al. 2019; Gebretsadik et al. 2022). This is related to the belief that meat is not good for the health of children less than 2 years of age and because parents perceive that young children are not ready for meat and other nondairy ASFs until they are older (Ali et al. 2011).

Some women worried that eating ASFs during pregnancy would make the baby bigger and lead to a possible obstructed labour. This study finding aligns with previous studies in Ethiopia that reported lower consumption of different foodstuffs, including ASFs, due to this concern, which needs intervention to address the local sociocultural norms (Zerfu et al. 2016; Demilew et al. 2020).

Fasting temporarily prohibits ASF consumption by Orthodox Christians and Muslims. This practice has adverse effects on the health and wellbeing of pregnant women and young children, particularly those who are Orthodox Christians because there are around 250 fasting days per year. Pregnant women do not eat ASFs during the fasting days, and children are obliged to start fasting at the age of seven and beyond. Moreover, the community restricts the provision of ASFs to all children during the fasting season because they fear contamination of cooking utensils. This finding is consistent with previous studies (McNamara and McKune 2018; Demilew et al. 2020).

The community trusted information from professionals and religious leaders. The existing supports to improve maternal and child nutrition were not adequate to bring desired effect in improving ASF consumption. Poor leadership, inadequate support, and mistrust of the community on the distribution mechanism of support were barriers to maternal and child nutrition programme implementation in the study area. The existing structure in the primary health care system, from the national to local kebele level, provides good opportunities for nutrition intervention implementation.

Respondents recommended financial support to start income‐generating activities, provision of livestock, provision of animal feed, and nutrition education to improve ASF consumption among children and pregnant and lactating women. The findings of this study have significant policy implications for the implementation of income generation programmes and livestock provision to poor households.

## 5 Conclusions

Pregnant and lactating women considered ASFs as food for holidays and societal events. Information from professionals and religious leaders was thought to be reliable and trusted, but the community said they distrusted nutrition information given by the health development army. The high cost of ASFs and financial constraints within households discouraged pregnant and lactating women and children from consuming ASFs. Family affluence, the availability of ASFs in the household, liking the taste of ASFs, and receiving nutrition education on ASFs were factors that facilitated the consumption of ASFs by children and pregnant and lactating women. Social and behaviour change communication should be designed through the existing health extension programmes and strengthened to increase children's and pregnant and lactating women's ASF consumption. Livelihood support mechanisms such as provision of initial capital, domestic animals, and animal feed could also increase ASF consumption among vulnerable groups.

## Author Contributions

Netsanet Fentahun, Valerie Flax and Yeshalem Mulugeta conceived the study, developed the study method, analysed the data, and drafted and revised the manuscript. All authors read and approved the final manuscript.

## Conflicts of Interest

The authors declare no conflicts of interest.

## Data Availability

The data that support the findings of this study are available on request from the corresponding author. The data are not publicly available due to privacy or ethical restrictions.

## References

[mcn70006-bib-0001] Abegaz, G. A. , I. W. Hassen , and B. Minten . 2018. “Consumption of Animal‐Source Foods in Ethiopia: Patterns, Changes, and Determinants.” ESSP Working Papers No 113. International Food Policy Research Institute (IFPRI).

[mcn70006-bib-0002] Ahmed, S. K. 2024. “How to Choose a Sampling Technique and Determine Sample Size for Research: A Simplified Guide for Researchers.” Oral Oncology Reports 12: 100662.

[mcn70006-bib-0003] Ali, D. , M. Tedla , A. Subandoro , A. Bamezai , R. Rawat , and P. Menon . 2011. Alive & Thrive Baseline Survey Report: Ethiopia. Alive & Thrive.

[mcn70006-bib-0004] Amugsi, D. A. , A. Lartey , E. Kimani‐Murage , and B. U. Mberu . 2016. “Women's Participation in Household Decision‐Making and Higher Dietary Diversity: Findings From Nationally Representative Data From Ghana.” Journal of Health, Population, and Nutrition 35: 1–8. 27245827 10.1186/s41043-016-0053-1 PMC5026004

[mcn70006-bib-0005] Ayana, G. , T. Hailu , D. Kuche , et al. 2017. “Linkages Between Health and Agriculture Sectors in Ethiopia: A Formative Research Study Exploring Barriers, Facilitators and Opportunities for Local Level Coordination to Deliver Nutritional Programmes and Services.” BMC Nutrition 3: 69. 32153848 10.1186/s40795-017-0189-4 PMC7050871

[mcn70006-bib-0006] Azzarri, C. , A. Zezza , B. Haile , and E. Cross . 2015. “Does Livestock Ownership Affect Animal Source Foods Consumption and Child Nutritional Status? Evidence From Rural Uganda.” The Journal of Development Studies 51, no. 8: 1034–1059.

[mcn70006-bib-0007] Bonis‐Profumo, G. , D. do Rosario Pereira , J. Brimblecombe , and N. Stacey . 2022. “Gender Relations in Livestock Production and Animal‐Source Food Acquisition and Consumption Among Smallholders in Rural Timor‐Leste: A Mixed‐Methods Exploration.” Journal of Rural Studies 89: 222–234.

[mcn70006-bib-0008] Bukachi, S. A. , M. Ngutu , A. W. Muthiru , A. Lépine , S. Kadiyala , and P. Domínguez‐Salas . 2022. “Gender and Sociocultural Factors in Animal Source Foods (ASFs) Access and Consumption in Lower‐Income Households in Urban Informal Settings of Nairobi, Kenya.” Journal of Health, Population, and Nutrition 41, no. 1: 30. 35818082 10.1186/s41043-022-00307-9 PMC9275060

[mcn70006-bib-0009] Connelly, L. M. 2016. “Trustworthiness in Qualitative Research.” Medsurg Nursing: Official Journal of the Academy of Medical‐Surgical Nurses 25, no. 6: 435–436. 30304614

[mcn70006-bib-0010] Daba, A. K. , M. Murimi , K. Abegaz , and D. Hailu . 2021. “Determinants and Constraints to Household‐Level Animal Source Food Consumption in Rural Communities of Ethiopia.” Journal of Nutritional Science 10: e58. 34422260 10.1017/jns.2021.52 PMC8358840

[mcn70006-bib-0011] Demilew, Y. M. , G. D. Alene , and T. Belachew . 2020. “Dietary Practices and Associated Factors Among Pregnant Women in West Gojjam Zone, Northwest Ethiopia.” BMC Pregnancy and Childbirth 20, no. 1: 18. 31906981 10.1186/s12884-019-2702-z PMC6945405

[mcn70006-bib-0012] Dror, D. K. , and L. H. Allen . 2011. “The Importance of Milk and Other Animal‐Source Foods for Children in Low‐Income Countries.” Food and Nutrition Bulletin 32, no. 3: 227–243. 22073797 10.1177/156482651103200307

[mcn70006-bib-0013] Eaton, J. C. , P. Rothpletz‐Puglia , M. R. Dreker , et al. 2019. “Effectiveness of Provision of Animal‐Source Foods for Supporting Optimal Growth and Development in Children 6 to 59 Months of Age.” Cochrane Database of Systematic Reviews 2019, no. 2: CD012818. 10.1002/14651858.CD012818.pub2. PMC6380771 30779870

[mcn70006-bib-0014] Fite, M. B. , A. K. Tura , T. A. Yadeta , L. Oljira , and K. T. Roba . 2022. “Consumption of Animal Source Food and Associated Factors Among Pregnant Women in Eastern Ethiopia: A Community‐Based Study.” PLoS ONE 17, no. 6: e0270250. 35714168 10.1371/journal.pone.0270250 PMC9205500

[mcn70006-bib-0015] Gebretsadik, G. G. , A. K. Adhanu , and A. Mulugeta . 2022. “Magnitude and Determinants of Animal Source Food Consumption Among Children Aged 6–23 Months in Ethiopia: Secondary Analysis of the 2016 Ethiopian Demographic and Health Survey.” BMC Public Health 22, no. 1: 453. 35255843 10.1186/s12889-022-12807-8 PMC8900383

[mcn70006-bib-0016] Gernand, A. D. , K. J. Schulze , C. P. Stewart , K. P. West , and P. Christian . 2016. “Micronutrient Deficiencies in Pregnancy Worldwide: Health Effects and Prevention.” Nature Reviews Endocrinology 12, no. 5: 274–289. 10.1038/nrendo.2016.37 PMC4927329 27032981

[mcn70006-bib-0017] Grace, D. , P. Domínguez Salas , and S. Alonso , et al. 2018. “The Influence of Livestock‐Derived Foods on Nutrition During the First 1,000 Days of Life.” ILRI Research Report.

[mcn70006-bib-0018] Haileselassie, M. , G. Redae , G. Berhe , et al. 2020. “Why Are Animal Source Foods Rarely Consumed by 6‐23 Months Old Children in Rural Communities of Northern Ethiopia? A Qualitative Study.” PLoS ONE 15, no. 1: e0225707. 31914130 10.1371/journal.pone.0225707 PMC6948827

[mcn70006-bib-0019] Hanley‐Cook, G. T. , J. Y. A. Tung , I. F. Sattamini , et al. 2020. “Minimum Dietary Diversity for Women of Reproductive Age (MDD‐W) Data Collection: Validity of the List‐Based and Open Recall Methods as Compared to Weighed Food Record.” Nutrients 12, no. 7: 2039. 32659995 10.3390/nu12072039 PMC7400839

[mcn70006-bib-0020] Headey, D. 2018. “Animal Sourced Foods and Child Nutrition in South Asia: Policy priorities.”.

[mcn70006-bib-0021] Headey, D. , K. Hirvonen , and J. Hoddinott . 2018. "Animal Sourced Foods and Child Stunting." American Journal of Agricultural Economics 100, no. 5: 1302–1319. 10.1093/ajae/aay053. PMC7734193 33343003

[mcn70006-bib-0022] Hsieh, H.‐F. , and S. E. Shannon . 2005. “Three Approaches to Qualitative Content Analysis.” Qualitative Health Research 15, no. 9: 1277–1288. 16204405 10.1177/1049732305276687

[mcn70006-bib-0023] Jager, J. , D. L. Putnick , and M. H. Bornstein . 2017. “II. More Than Just Convenient: The Scientific Merits of Homogeneous Convenience Samples.” Monographs of the Society for Research in Child Development 82, no. 2: 13–30. 28475254 10.1111/mono.12296 PMC5606225

[mcn70006-bib-0024] Jin, M. , and L. L. Iannotti . 2014. “Livestock Production, Animal Source Food Intake, and Young Child Growth: The Role of Gender for Ensuring Nutrition Impacts.” Social Science & Medicine (1982) 105: 16–21. 24606793 10.1016/j.socscimed.2014.01.001

[mcn70006-bib-0025] Kim, S. S. , P. H. Nguyen , Y. Yohannes , et al. 2019. “Behavior Change Interventions Delivered Through Interpersonal Communication, Agricultural Activities, Community Mobilization, and Mass Media Increase Complementary Feeding Practices and Reduce Child Stunting in Ethiopia.” Journal of Nutrition 149, no. 8: 1470–1481. 31165869 10.1093/jn/nxz087 PMC6686053

[mcn70006-bib-0026] Kim, S. S. , R. Rawat , E. M. Mwangi , et al. 2016. “Exposure to Large‐Scale Social and Behavior Change Communication Interventions Is Associated With Improvements in Infant and Young Child Feeding Practices in Ethiopia.” PLoS ONE 11, no. 10: e0164800. 27755586 10.1371/journal.pone.0164800 PMC5068829

[mcn70006-bib-0027] McNamara, K. , and S. McKune . 2018. “Looking Beyond Productivity: Barriers to Animal Source Food Consumption in Ethiopia.”

[mcn70006-bib-0028] Melse‐Boonstra, A. 2020. “Bioavailability of Micronutrients From Nutrient‐Dense Whole Foods: Zooming in on Dairy, Vegetables, and Fruits.” Frontiers in Nutrition 7: 101. 32793622 10.3389/fnut.2020.00101 PMC7393990

[mcn70006-bib-0029] Miller, V. , J. Reedy , F. Cudhea , et al. 2022. “Global, Regional, and National Consumption of Animal‐Source Foods Between 1990 and 2018: Findings From the Global Dietary Database.” The Lancet Planetary Health 6, no. 3: e243–e256. 35278390 10.1016/S2542-5196(21)00352-1 PMC8926870

[mcn70006-bib-0030] Nana, A. , and T. Zema . 2018. “Dietary Practices and Associated Factors during Pregnancy in Northwestern Ethiopia.” BMC Pregnancy and Childbirth 18, no. 1: 183. 29801471 10.1186/s12884-018-1822-1 PMC5970492

[mcn70006-bib-0031] Neumann, C. G. , S. P. Murphy , C. Gewa , M. Grillenberger , and N. O. Bwibo . 2007. “Meat Supplementation Improves Growth, Cognitive, and Behavioral Outcomes in Kenyan Children.” Journal of Nutrition 137, no. 4: 1119–1123. 17374691 10.1093/jn/137.4.1119

[mcn70006-bib-0032] Potts, K. S. , A. Mulugeta , and A. N. Bazzano . 2019. “Animal Source Food Consumption in Young Children From Four Regions of Ethiopia: Association With Religion, Livelihood, and Participation in the Productive Safety Net Program.” Nutrients 11, no. 2: 354. 30743994 10.3390/nu11020354 PMC6413062

[mcn70006-bib-0033] Schmid, A. , and B. Walther . 2013. “Natural Vitamin D Content in Animal Products.” Advances in Nutrition 4, no. 4: 453–462. 23858093 10.3945/an.113.003780 PMC3941824

[mcn70006-bib-0034] Tafere, K. , and I. W. Hassen . 2012. “Consumption Patterns of Livestock Products in Ethiopia: Elasticity Estimates Using HICES (2004/05) Data.”

[mcn70006-bib-0035] World Health Organization . 2014. *Comprehensive Implementation Plan on Maternal, Infant and Young Child Nutrition*. World Health Organization Technical document, WHO/NMH/NHD/14.1. 10.3945/an.114.007781 PMC4288273 25593153

[mcn70006-bib-0036] World Health Organization . 2023. *WHO Guideline for Complementary Feeding of Infants and Young Children 6‐23 Months of Age*. World Health Organization. 37871145

[mcn70006-bib-0037] Wong, J. T. , B. Bagnol , H. Grieve , J. B. da Costa Jong , M. Li , and R. G. Alders . 2018. “Factors Influencing Animal‐Source Food Consumption in Timor‐Leste.” Food Security 10: 741–762.

[mcn70006-bib-0038] Zerfu, T. A. , M. Umeta , and K. Baye . 2016. “Dietary Habits, Food Taboos, and Perceptions Towards Weight Gain During Pregnancy in Arsi, Rural Central Ethiopia: A Qualitative Cross‐Sectional Study.” Journal of Health, Population, and Nutrition 35, no. 1: 22. 27456151 10.1186/s41043-016-0059-8 PMC5025964

